# Serum Calprotectin Demonstrates Moderate Diagnostic Performance for Detecting Periprosthetic Joint Infection in Hip and Knee Arthroplasty

**DOI:** 10.1016/j.artd.2026.102194

**Published:** 2026-11-03

**Authors:** Morlin Hannah Schelling, Jan Schwarze, Georg Gosheger, Kristian Nikolaus Schneider, Sebastian Bockholt, Christoph Theil

**Affiliations:** Department of Orthopedics and Tumor Orthopedics, Muenster University Hospital, Muenster, Germany

**Keywords:** Periprosthetic joint infection, Calprotectin, CRP, Interleukin-6, Arthroplasty, Biomarker

## Abstract

**Background:**

Periprosthetic joint infection (PJI) is a serious complication after total hip and knee arthroplasty, with limited accuracy of conventional biomarkers. This study assessed serum calprotectin (CP) alongside established markers in revision arthroplasty.

**Methods:**

This retrospective single-center study included 399 patients (232 total knee arthroplasty, 167 total hip arthroplasty). Based on Musculoskeletal Infection Society 2018 criteria, 132 patients were diagnosed with PJI and 267 were classified as non-PJI. All patients underwent serum CP, C-reactive protein (CRP), and interleukin-6 (IL-6) testing; microbiological analyses were performed when indicated.

**Results:**

Serum CP was significantly higher in PJI than aseptic revisions (median 11,700 ng/mL [interquartile range 8080-18,675] vs 5,440 ng/mL [interquartile range 3600-8300], *P* < .001). The optimal CP threshold for PJI was 8,330 ng/mL (area under the curve = 0.790, 95% CI, 0.741-0.838), providing a sensitivity of 65.2% (95% CI, 56.7-72.7%) and a specificity of 79.8% (95% CI, 74.6-84.2%). CRP and (IL)-6 showed higher diagnostic performance, with sensitivities of 69.0% (95% CI, 60.6-76.2%) and 73.6% (95% CI, 65.4-80.5%) and specificities of 88.4% (95% CI, 83.9-91.6%) and 85.0% (95% CI, 80.2-88.8%). Serum CP levels were not significantly influenced by joint type, sex, or age. In 8 patients with PJI, serum CP was above the cohort-derived threshold while both CRP and IL-6 remained below their respective thresholds. Conversely, in 10 cases, serum CP remained below the threshold despite elevated CRP and IL-6.

**Conclusions:**

Serum calprotectin demonstrated moderate diagnostic performance and may may complement conventional serum markers in selected cases in which conventional serum markers are inconclusive.

## Introduction

Periprosthetic joint infection (PJI) is a serious complication following total hip (THA) and knee arthroplasty (TKA) which occurs in approximately 1-2% of primary arthroplasties [1], [2], [3]. Following revision procedures, the infection rate rises to 5-15% [2], and multiply revised implants face increasing risk for PJI with every revision performed [4]. With increasing procedure volumes worldwide, the burden of PJI is projected to further increase in coming years [5], [6], [7].

Over the last 15 years, several diagnostic algorithms and criteria have evolved in order to provide for a diagnostic standard [8]. Frequently used are those established by the Musculoskeletal Infection Society (MSIS) and the European Bone and Joint Infection Society that in addition to clinical findings such as fistula and microbiology findings, rely on different serum and synovial fluid parameters to establish the diagnosis [7], [8], [9], [10].

While several synovial fluid markers, most commonly the synovial fluid white blood cell count and percentage of polymorphonuclear cells offer good diagnostic accuracy [11], they are usually combined with systemic, serum inflammatory markers usually C-reactive protein (CRP). However, noninfectious inflammation may lead to false-positive synovial fluid findings [8], [12], [13], [14] and there is the risk of a “dry tap.” Overall, none of these markers achieves perfect diagnostic accuracy, and no single gold standard test is available for the diagnosis of PJI [5], [8], [15].

While serum CRP is the most common blood marker, it has been shown to misdiagnose low-grade infections, as approximately one-third of patients have a normal or only minimally elevated CRP [8], [14], [15]. Additionally, serum parameters with high specificity such as interleukin-6 (IL-6) [5], [8], [11], [16] have been used as an adjunct test but are subject to similar limitations [17]. These issues prompt the search for novel serum markers. One promising marker is serum calprotectin (CP) [8].

CP is a heterodimer composed of S100A8 and S100A9 proteins, belonging to the family of calcium-binding proteins. Commonly found in the cytosol of neutrophils and a few other cell types, it is involved in inflammatory responses. CP has been established as a parameter for inflammatory bowel disease; however, its role in diagnosing PJI remains exploratory [18]. Most existing studies have examined synovial CP for the diagnosis of PJI [12], [19], [20], with only a few exploring serum CP using various assays and methodologies.

Given these characteristics, this study aims to further evaluate the reliability of serum CP for diagnosing PJI in THA and TKA. The analysis focused on determining an optimal diagnostic threshold for CP, assessing its sensitivity and specificity, and comparing its performance with established serum biomarkers, including CRP and IL-6. In addition, potential factors influencing serum CP levels, including age, sex, and joint type, (THA vs TKA), were explored in an exploratory analysis to better characterize its clinical applicability.

## Material and methods

This study was approved by the institutional review board of the authors’ institution (ethics committee of the University of Muenster, ref. no. 2019-666-fS). The study was conducted in accordance with the Declaration of Helsinki, and written consent was given by all participants. No external funding was received.

We retrospectively identified 399 consecutive patients with painful THA or TKA who underwent serum CP testing between October 2019 and December 2022 as part of the clinical diagnostic evaluation for suspected PJI at our institution. All patients were evaluated for chronic symptoms persisting for at least 6 weeks. Patients were evaluated because of persistent pain and/or other clinical findings that prompted the treating physician to consider PJI. The cohort was not restricted to patients undergoing revision surgery and therefore included patients across a range of clinical suspicions for PJI. The decision to perform joint aspiration, further microbiological testing, or revision surgery was made by the treating clinical team based on the individual clinical presentation and available diagnostic findings and was not determined by the study protocol.

Of the 399 patients, 130 underwent revision surgery and 269 did not. Among patients who underwent revision surgery, 105 were classified as having PJI and 25 as not having PJI. Among patients who did not undergo revision surgery, 27 were classified as having PJI and 242 as not having PJI based on the final clinical diagnosis documented in the patient records and the available diagnostic findings.

The cohort included 58.1% (232/399) of patients with TKA and 41.9% (167/399) of patients with THA. Among patients who underwent revision surgery, 105 were diagnosed with PJI and 25 were classified as noninfected. Among those who did not undergo revision surgery, 27 patients were diagnosed with PJI based on clinical, laboratory, and microbiological criteria, while 242 were considered not infected. Patients presenting with suspected PJI underwent clinical evaluation and diagnostic workup including serum CRP and IL-6 measurements and, when clinically indicated, synovial fluid, and microbiological analyses. The final clinical diagnosis documented in the patient records was used for retrospective study classification. This diagnosis had been established in routine clinical practice based on the 2018 MSIS/International Consensus Meeting diagnostic framework and the available clinical, laboratory, synovial, microbiological, and intraoperative findings. The individual MSIS/International Consensus Meeting criteria were not separately collected as study variables or retrospectively readjudicated for the purpose of this study.

Overall, 132 patients (33.1%) were classified as having PJI and 267 patients (66.9%) as not having PJI.

In this study, 52.9% (211/399) of patients were women and 47.1% (188/399) of patients were men, with a median age of 68.27 years (interquartile range [IQR], 60.69-77.96), and 49.6% (n = 198) of patients were aged ≥70 years. The median age did not differ significantly between women and men (68.01 years [IQR, 60.31-77.17] vs 71.84 years [IQR, 61.53-78.50], respectively; *P* = .147) (Table 1), (Fig. 1).

**Table 1:** Baseline characteristics of patients with and without periprosthetic joint infection.

Characteristic	PJI (n = 132)	Non-PJI (n = 267)	*P* value
Age, median (IQR), years	73.41 (65.24–79.37)	68.27 (58.51–76.13)	<.001
Sex, n (%)			
Male	61 (46.2)	127 (47.6)	—
Female	71 (53.8)	140 (52.4)	—
Affected joint, n (%)			
Knee	76 (57.6)	156 (58.4)	—
Hip	56 (42.4)	111 (41.6)	—

**Figure 1:**
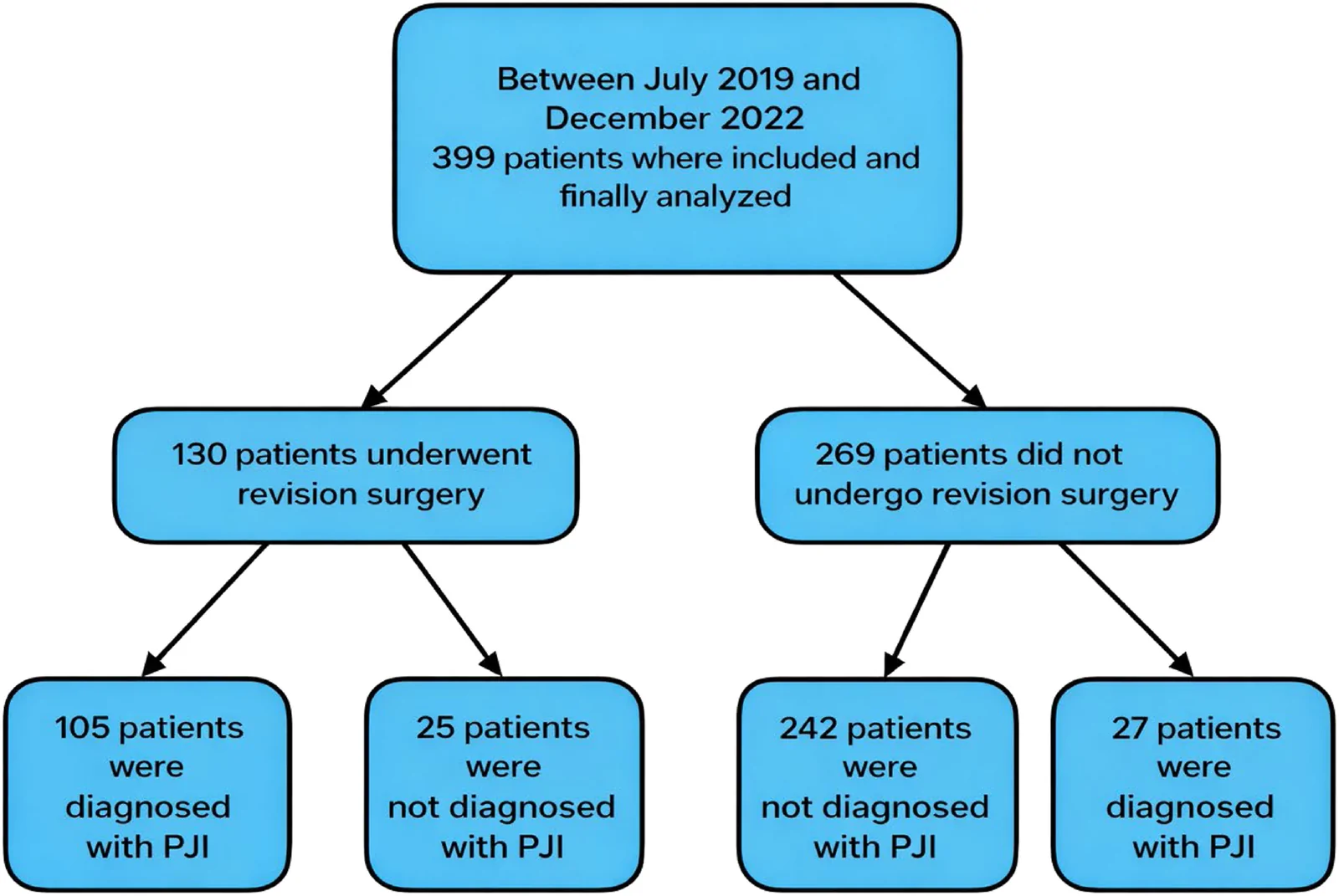
STROBE (Strengthening the reporting of observational studies in epidemiology) diagram of patients shows study design.

Serum CP was measured using the fCAL turbo immunoturbidimetric assay (BÜHLMANN Laboratories AG, Schönenbuch, Switzerland), which was originally developed for fecal CP measurement. For serum analysis in the present study, samples were diluted 1:10 before analysis on a Cobas c502 clinical chemistry analyzer (Roche Diagnostics GmbH, Mannheim, Germany). Serum CP concentrations are reported in nanograms per milliliter (ng/ml). The assay has not been formally validated by the manufacturer for serum samples.

All patients underwent a clinical diagnostic workup including the analysis of serum CRP and serum IL-6. When clinically indicated, synovial fluid was analyzed for leukocyte count, percentage of synovial neutrophils and microbiological culture. Intraoperative tissue samples were evaluated. The number of intraoperative tissue samples was determined by the treating surgeon based on intraoperative findings, as no predefined minimum or maximum number of samples was mandated by the study protocol.

Synovial fluid and intraoperative tissue samples were cultured for at least 14 days on Columbia blood agar, chocolate agar, and Schaedler agar for microbiological testing. Single positive cultures were considered contaminated if all other findings were not suggestive of PJI in accordance with the criteria proposed by the MSIS [11].

Patients who had undergone surgery within the last 4 weeks, as well as those with chronic systemic inflammatory conditions such as rheumatoid arthritis, malignancies, and confirmed inflammatory diseases of other organs (eg, inflammatory bowel diseases, urinary tract infection and pneumonia) were not screened using serum CP testing.

Microbiological assessment was performed in 322 of 399 patients as part of the clinical diagnostic pathway and included synovial fluid aspiration, intraoperative tissue sampling, and both. Synovial aspiration was not routinely performed in 77 patients with a low preoperative probability of PJI, particularly when serum inflammatory markers were not elevated or a recent aspiration had already been performed at an external institution, providing the majority of parameters available for preoperative MSIS classification. Importantly, all 132 patients ultimately classified as having PJI underwent microbiological sampling, either by synovial aspiration, intraoperative tissue sampling, or both, although microbiological cultures were not positive in all cases.

Microbiological testing yielded positive results in 117 patients. Twenty-four of these patients (20.5%) had polymicrobial cultures. The most frequently identified organisms were coagulase-negative *Staphylococcus* spp. (54.7%; 64/117), *Staphylococcus aureus* (8.5%; 10/117), gram-negative bacteria (22.2%; 26/117), *Streptococcus* spp. (11.9%; 14/117), and *Cutibacterium* spp. (11.9%; 14/117). Additionally, one (0.9%) culture of *Candida spp*. and 11.1% (13/117) cultures with microbes not fitting into the above categories were identified. Notably, 7.6% (10/132) patients later diagnosed with PJI had negative microbiological results from intraoperative samples, and 18.2% (24/132) had culture-negative synovial fluid aspirations.

Data collection and statistical analysis were conducted using Microsoft Excel (Microsoft Corporation, Redmond, WA, USA) and the Statistical Package for the Social Sciences (SPSS) version 31 (IBM Corporation, Armonk, NY, USA). Patient data were pseudonymized prior to analysis. Descriptive statistics were used and the Shapiro–Wilk and Kolmogorov–Smirnov tests were performed to assess data distribution. For normally distributed data, means and ranges were reported, whereas medians and IQRs were reported for non-normally distributed data. The Mann–Whitney *U*-test was used for comparisons of non-normally distributed continuous variables. Categorical variables were presented as frequencies and compared using *chi*-squared tests in contingency tables.

Receiver operating characteristic (ROC) analysis was employed to evaluate diagnostic performance, with the area under the curve (AUC) reported alongside 95% confidence intervals (CIs). Youden's index was utilized to determine the optimal cutoff values for serum CP, CRP, and IL-6. For exploratory subgroup analyses, optimal cut-off values were additionally determined separately for THA and TKA using Youden’s index. No internal or external validation of the derived cutoff values was performed. Spearman’s rank correlation coefficient (Spearman’s rho) was used to assess the strength and direction of the monotonic relationship between the variables. Statistical significance was defined as *P* ≤ .05.

Sensitivity and specificity were classified as high (≥90%), moderate (70-89%), or low (<70%). AUC values were interpreted as excellent (≥0.90), good (0.80-0.89), moderate (0.70-0.79), or poor (<0.70). These criteria were applied consistently across all markers analyzed in the study.

## Results

The median serum CP was significantly higher in patients with PJI than patients without PJI (11,700 ng/ml [IQR, 8080-18,675] vs 5440 ng/ml [IQR, 3600-8300]; *P* < .001). Among patients undergoing revision THA, those with PJI had significantly higher median serum CP levels than patients without PJI (12,660 ng/ml [IQR, 8100-17,880] vs 5020 ng/ml [IQR, 3380-8680]; *P* < .001). Similar results were found for revision TKA (11,060 ng/ml [IQR, 8190-18,810] vs 5470 ng/ml [IQR, 3880-8140]; *P* < .001) (Fig. 2).

**Figure 2:**
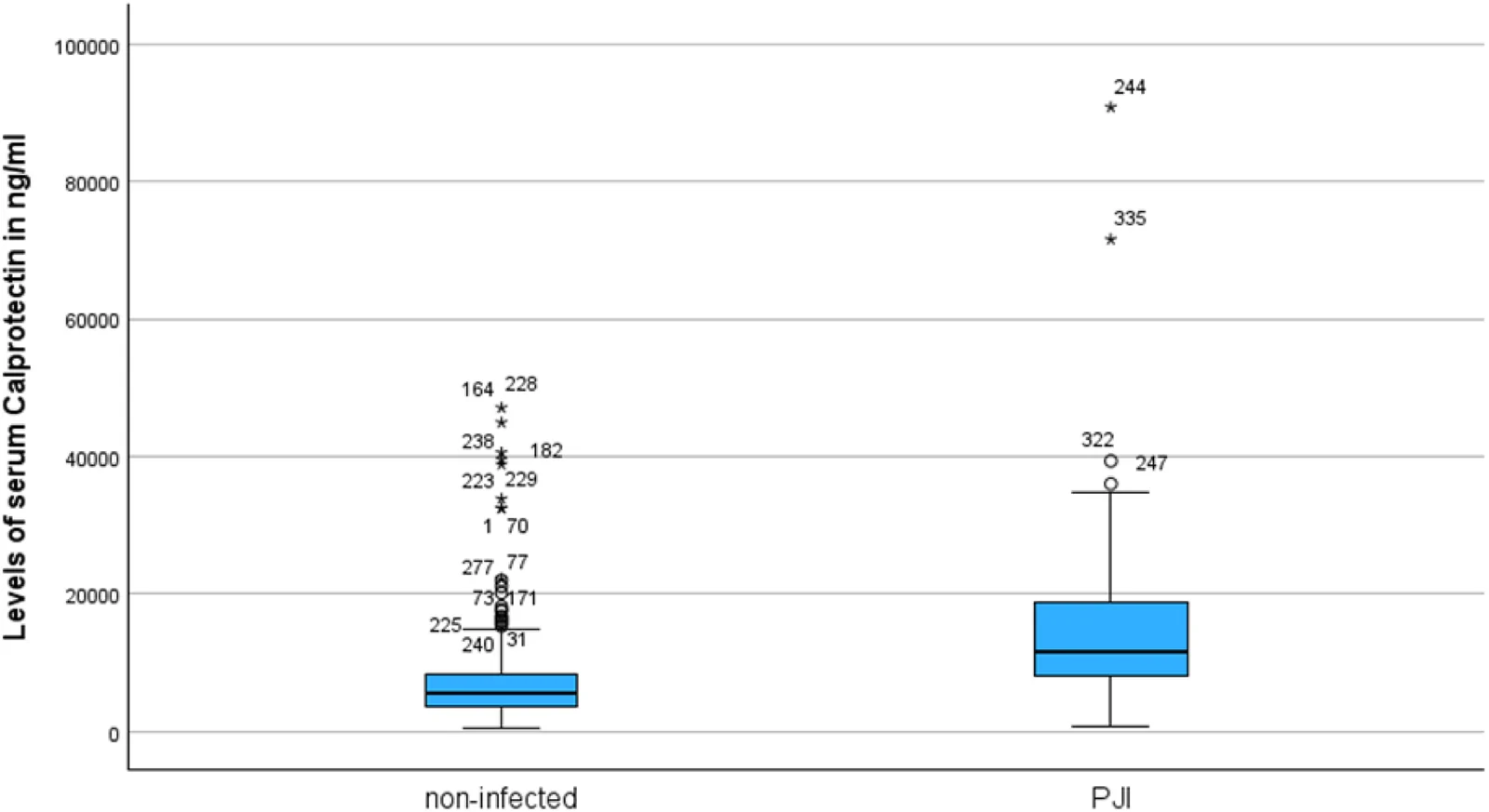
Levels of serum CP in patients with PJI and noninfected cases.

The ROC analysis yielded an AUC of 0.790 (95% CI 0.741-0.838) for serum CP (*P* < .001). The calculated optimal threshold for serum CP was 8330 ng/mL. Using this cut-off value, serum CP levels were above the threshold in 65.2% (86/132) of patients with PJI and in 20.2% (54/267) of patients without PJI. This cut-off value yielded a sensitivity of 65.2% (95% CI, 56.7-72.7%), a specificity of 79.8% (95% CI, 74.6-84.2%), a positive predictive value (PPV) of 61.4% (95% CI, 53.2-69.1%), and a negative predictive value (NPV) of 82.2% (95% CI, 77.1-86.4%) for serum CP.

Sensitivity analyses restricted to patients with microbiological sampling (n = 322) and to those undergoing revision surgery with intraoperative tissue sampling (n = 130) yielded diagnostic performance estimates for serum CP that were broadly comparable to those of the primary analysis. In patients with microbiological sampling (n = 322), the diagnostic performance of serum CP remained comparable to that of the primary analysis, with an AUC of 0.790 (95% CI, 0.739-0.842), sensitivity of 65.2% (95% CI, 56.7-72.7%), and specificity of 80.0% (95% CI, 73.7-85.1%). Similarly, among patients who underwent revision surgery with intraoperative tissue sampling (n = 130), serum CP demonstrated an AUC of 0.800 (95% CI, 0.691-0.908), sensitivity of 63.8% (95% CI, 54.3-72.4%), and specificity of 84.0% (95% CI, 65.3-93.6%) (Table 2) (Fig. 3).

**Table 2:** Diagnostic performance of serum calprotectin, C-reactive protein, and interleukin-6 for the diagnosis of periprosthetic joint infection.

Analysis	Biomarker	AUC (95% CI)	Sensitivity % (95% CI)	Specificity % (95% CI)	PPV % (95% CI)	NPV % (95% CI)
Overall (n = 399)	CP	0.790 (0.741-0.838)	65.2 (56.7-72.7)	79.8 (74.6-84.2)	61.4 (53.2-69.1)	82.2 (77.1-86.4)
	CRP	0.853 (0.811-0.896)	69.0 (60.6-76.2)	88.4 (83.9-91.6)	74.8 (66.2-81.5)	85.1 (80.4-88.8)
	IL-6	0.858 (0.817-0.900)	73.6 (65.4-80.5)	85.0 (80.2-88.8)	70.9 (62.7-77.9)	86.7 (81.9-90.3)
Microbiological sampling (n = 322)	CP	0.790 (0.739-0.842)	65.2 (56.7-72.7)	80.0 (73.7-85.1)	69.4 (60.8-76.8)	76.8 (70.4-82.1)
	CRP	0.849 (0.804-0.894)	64.1 (55.6-71.8)	88.9 (83.6-92.9)	80.0 (71.1-87.0)	78.1 (72.1-83.4)
	IL-6	0.856 (0.812-0.900)	73.6 (65.4-80.5)	84.3 (78.5-88.8)	76.6 (68.1-83.7)	82.1 (76.0-87.0)
Revision surgery with intraoperative sampling (n = 130)	CP	0.800 (0.691-0.908)	63.8 (54.3-72.4)	84.0 (65.3-93.6)	94.4 (86.4-97.8)	35.6 (24.6-48.3)
	CRP	0.841 (0.756-0.925)	62.5 (53.0-71.2)	88.0 (69.7-95.6)	95.6 (87.9-98.5)	36.1 (25.5-48.7)
	IL-6	0.817 (0.736-0.897)	70.6 (61.0-78.6)	85.7 (65.0-95.7)	96.0 (89.0-98.6)	37.5 (25.2-51.8)

Cut-off values for the primary analysis were determined using Youden’s index in the overall cohort and were subsequently applied to the sensitivity analysis subgroups. Cut-off values were not re-estimated within the sensitivity-analysis subgroups. No internal or external validation was performed.

**Figure 3:**
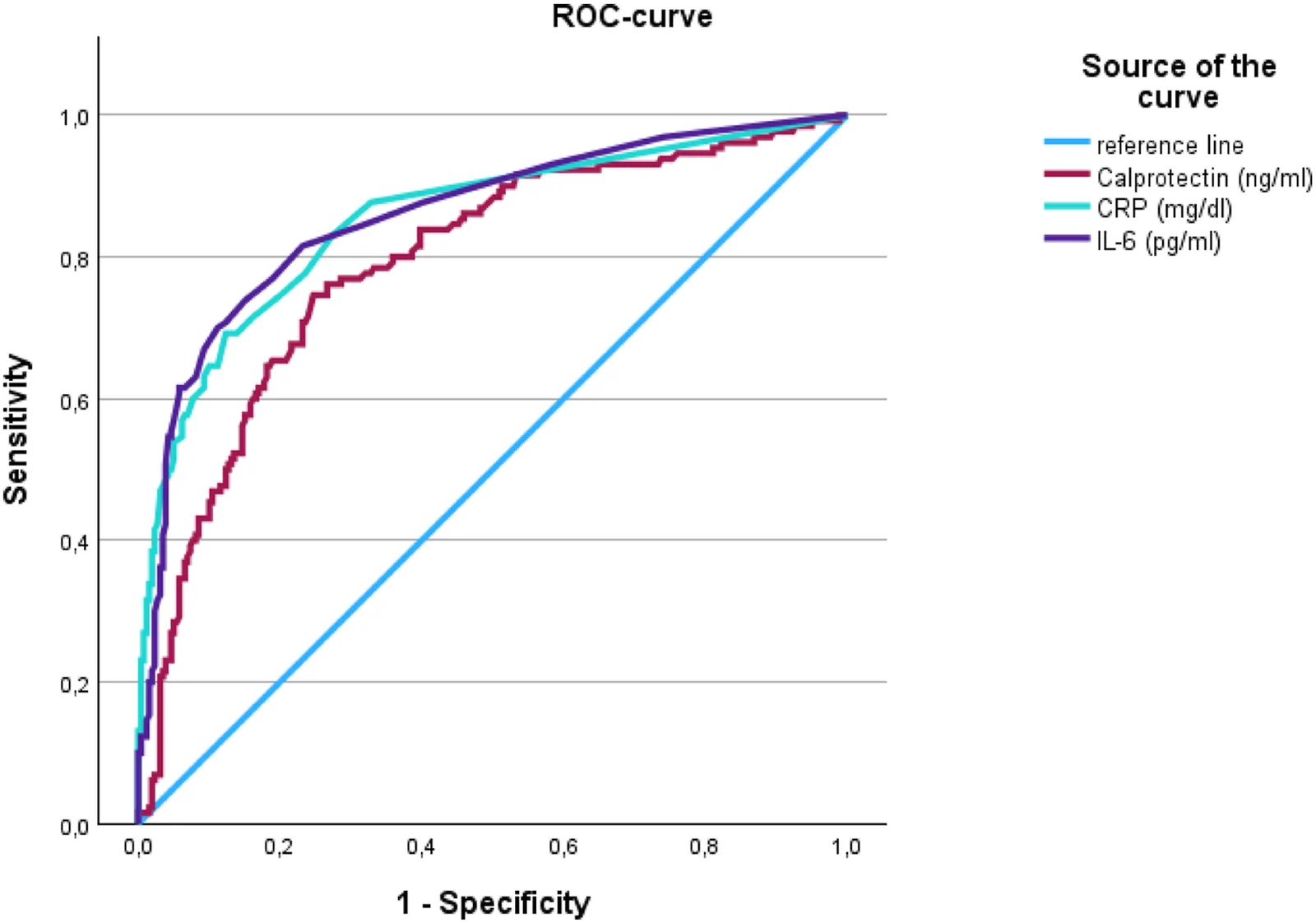
ROC curve of the levels of CP, CRP, and IL-6 for diagnosing a PJI using the estimated cut-off-values.

Separate analyses for patients with TKA and THA found similar results. In patients with THA ROC analyses revealed an AUC of 0.776 (95%, Cl 0.702-0.851, *P* < .001) resulting in an estimated optimal cut-off value of 8360 ng/ml for serum CP. Using the estimated cut-off values, serum CP levels were above the respective thresholds in 73.2% (41/56) of patients with PJI and in 24.3% (27/111) of patients without PJI, resulting in a sensitivity of 73.2% and specificity of 75.7%, with a PPV of 60.3% and an NPV of 84.8%.

For patients with TKAs the AUC was 0.789 (95%, CI 0.733-0.863, *P* < .001) with an estimated optimal cut-off value of 8260 ng/ml for serum CP. Using the estimated cut-off value, serum CP levels were above the threshold in 75.0% (57/76) of patients with PJI and in 24.4% (38/156) of patients without PJI, resulting in a sensitivity of 75% and specificity of 75.6% with a PPV of 60% and an NPV of 86.1% (Table 3).

**Table 3:** Serum biomarker levels and diagnostic performance according to arthroplasty type.

Arthroplasty type	Parameter	CP (ng/mL)	CRP (mg/dL)	IL-6 (pg/mL)
THA	Median (IQR)	6960 (3940-13,020)	0.5 (0.5-1.95)	5.0 (3.0-13.0)
	Cutoff value	8360	0.95	6.5
	Sensitivity, %	73.2 (60.4-83.0)	61.8 (48.6-73.4)	70.9 (57.8-81.4)
	Specificity, %	75.7 (66.9-82.7)	89.9 (82.9-94.1)	81.7 (73.4-87.7)
	PPV, %	60.3 (48.4-71.1)	75.6 (62.1-85.2)	66.1 (53.4-76.6)
	NPV, %	84.8 (76.5-90.6)	82.4 (75.0-87.7)	84.8 (77.0-90.2)
TKA	Median (IQR)	6680 (4430-10,635)	0.6 (0.5-1.975)	6.0 (3.0-16.0)
	Cut-off value	8260	1.35	7.5
	Sensitivity, %	75.0 (64.2-83.4)	65.8 (54.6-75.3)	75.7 (64.8-84.0)
	Specificity, %	75.6 (68.3-81.7)	89.7 (84.0-93.6)	87.4 (81.2-91.8)
	PPV, %	60.0 (49.9-69.3)	75.8 (64.2-84.6)	74.7 (63.8-83.1)
	NPV, %	86.1 (79.4-90.9)	84.3 (78.0-89.0)	88.0 (81.8-92.3)

Values are presented as median or percentage, as appropriate. Values for sensitivity, specificity, PPV, and NPV are presented with 95% CIs. Cut-off values were determined separately for THA and TKA using Youden’s index.

No significant differences in median serum CP levels were observed between patients with THA and TKA (6960 ng/ml, IQR 3940-13,020 ng/ml vs 6680 ng/ml, IQR 4430-10,635 ng/ml, *P* = .949). Additionally, serum CP values did not significantly differ between women and men. (6660 ng/ml, IQR 4160-11,560 ng/ml, *P* = .880).

The median age differed significantly between patients without and with PJI (68.27 years [IQR, 58.51-76.13] vs 73.41 years [IQR, 65.24-79.37], respectively; *P* < .001). However, age did not significantly correlate with serum CP levels (rho = 0.075, *P* = .134). Exploratory analysis by age subgroup showed that patients aged ≥70 years had a median serum CP of 7120 ng/ml (IQR 4630-12,250), compared with 6480 ng/ml (IQR 3970-11,160) in patients <70 years, with no significant difference (*P* = .243). Similarly to serum CP, CRP and IL-6 differed between patients who had PJI and those who did not. In patients without PJI, the median CRP level was 0.5 mg/dL (IQR, 0.5-0.7), compared with 3.2 mg/dL (IQR, 0.8-8.0) in patients with PJI (*P* < .001). For IL-6, the median level was 4.0 pg/ml (IQR 2.0-6.0 pg/ml, *P* < .001) for patients without PJI vs 21.0 pg/ml (IQR 8.0-43.5 pg/ml, *P* < .001) for patients with PJI.

Similar to serum CP, optimal threshold values for serum CRP and IL-6 were determined using ROC analysis, with an AUC of 0.853 (95% CI, 0.811-0.896) for CRP and 0.858 (95% CI, 0.817-0.900) for IL-6 (both *P* < .001). Optimal cut-off values for diagnosing PJI were 1.55 mg/dL for CRP and 8.5 pg/mL for IL-6. At these thresholds, serum CRP demonstrated a sensitivity of 69.0% (95% CI, 60.6-76.2%), specificity of 88.4% (95% CI, 83.9-91.6%), PPV of 74.8% (95% CI, 66.2-81.5%), and NPV of 85.1% (95% CI, 80.4-88.8%). Serum IL-6 demonstrated a sensitivity of 73.6% (95% CI, 65.4-80.5%), specificity of 85.0% (95% CI, 80.2-88.8%), PPV of 70.9% (95% CI, 62.7-77.9%), and NPV of 86.7% (95% CI, 81.9-90.3%) (Fig. 4, Fig. 5).

**Figure 4:**
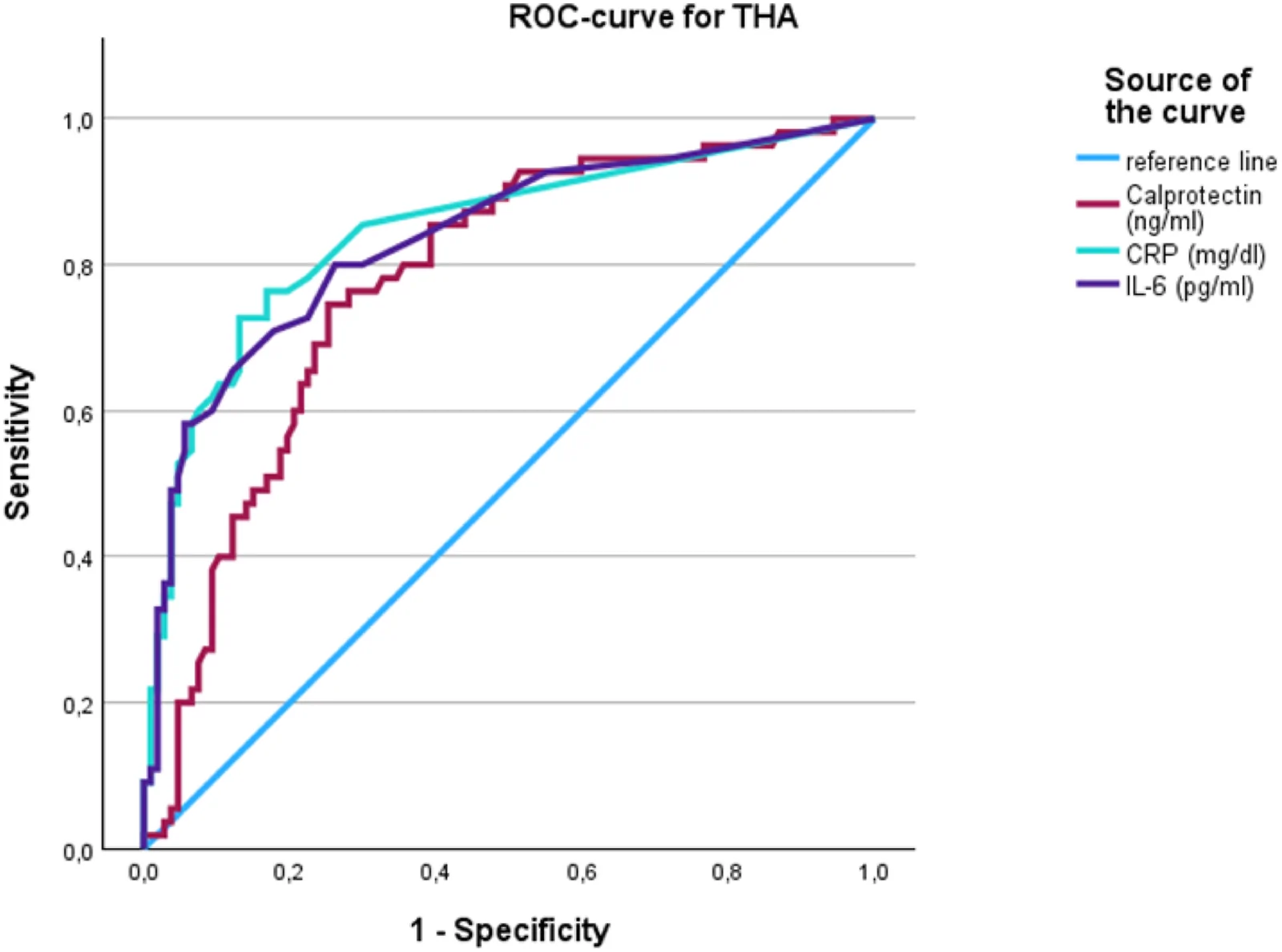
ROC curve of the serum levels of CP, CRP, and IL-6 for THA using the estimated cut-off values.

**Figure 5:**
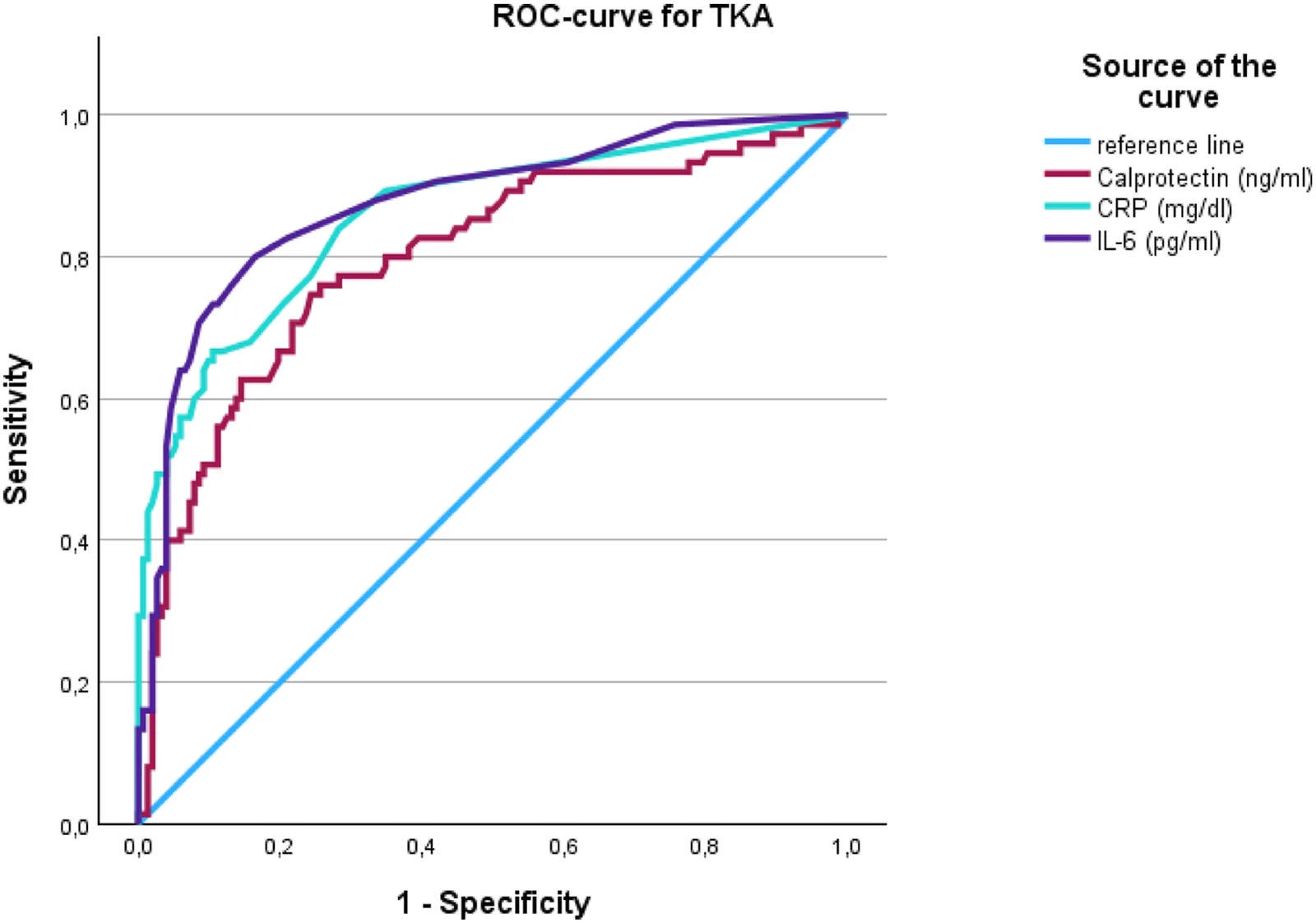
ROC curve of the serum levels of CP, CRP, and IL-6 for TKA using the estimated cut-off values.

Like serum CRP, there was no significant difference in median IL-6 and CRP levels between patients with THA and TKA (0.5 pg/mL [IQR, 0.5-1.95] vs 0.6 pg/mL [IQR, 0.5-1.975], *P* = .589 for CRP; 5.0 mg/dL [IQR, 3.0-13.0] vs 6.0 mg/dL [IQR, 3.0-16.0], *P* = .475 for IL-6).

No significant differences were found in IL-6 and CRP levels between the sexes (0.6 mg/dL [IQR, 0.5-1.9] vs 0.5 mg/dL [IQR, 0.5-1.95], *P* = .971 for CRP; 5.0 pg/mL [IQR, 3.0-13.5] vs 6.0 pg/mL [IQR, 4.0-13.5], *P* = .093 for IL-6).

In 8 patients with PJI, serum CP was positive (median, 16,670 ng/mL [IQR, 10,700-18,635]), whereas serum CRP (median, 0.7 mg/dL [IQR, 0.525-0.975]) and IL-6 (median, 4.0 pg/mL [IQR, 4.0-5.75]) were negative.

In 10 cases, patients with PJI had serum CP levels below the threshold while CRP and IL-6 were elevated.

## Discussion

As the global population ages, the need for need joint replacement surgery including THA and TKA, rises with an estimated increase of 23% and 45% until 2040 [21]. With this development and the high clinical and economic burden [5] associated with PJI, its early and accurate diagnosis becomes even more important. This study evaluated the diagnostic performance of serum CP in a real-world cohort of patients with painful hip or knee arthroplasties undergoing evaluation for suspected PJI and compared its performance with established serum biomarkers CRP and IL-6.

In our cohort, serum CP concentrations were significantly higher in patients with PJI than in those without PJI, and serum CP demonstrated moderate diagnostic discrimination (AUC 0.790). Using a cohort-derived cutoff of 8330 ng/ml, sensitivity was 65.2% and specificity was 79.8%. Thus, serum CP showed measurable diagnostic value but did not outperform CRP or IL-6, which demonstrated higher overall discriminatory performance. These findings therefore do not support serum CP as a superior standalone biomarker for PJI. Rather, they suggest that CP may provide complementary information in selected patients.

There have been smaller studies investigating serum CP that, similar to this study, found potential for diagnosing PJI.

One study recruiting fewer patients (n = 81) found a sensitivity of 81% and specificity of 91% at a serum CP cutoff of 9910 ng/ml, using the same immunoturbidimetric assay and a similar methodology [22]. The lower diagnostic performance observed in our larger cohort may reflect differences in patient selection, disease spectrum, comorbidities, and clinical heterogeneity. In contrast to smaller, more selected cohorts, our study included a broad real-world population undergoing evaluation for suspected PJI. This may provide a more clinically representative estimate of biomarker performance, although it may also result in lower apparent diagnostic accuracy.

Another study examined serum CP among other serum and synovial markers using a different immunoassay. The estimated sensitivity and specificity yielded at 93.3% and 87.5% with a cut-off value of 1 mg/l. However, this study only evaluated patients with chronic PJI at a small number (n = 45) among control groups [23]. Patients with early failure, revision arthroplasty and overweight or underweight patients were excluded. The higher diagnostic performance reported in that study may therefore partly reflect its more narrowly defined patient population and the use of a different assay. Direct comparison of absolute CP concentrations and cut-off values between studies should consequently be made with caution, particularly because different analytical platforms and serum preparation procedures were used.

The study identified an optimal serum CP threshold of 8330 ng/ml for diagnosing PJI. While the sensitivity of serum CP at this threshold (65.2%) was not particularly high, the specificity of 79.8% was encouraging. The NPV of 82.2% indicates that a negative serum CP result alone would not reliably exclude PJI. Therefore, serum CP should not be interpreted as a standalone rule-out test, particularly in patients with persistent clinical suspicion. Rather, CP should be interpreted in conjunction with clinical findings, established serum biomarkers, and other diagnostic investigations.

The cutoff identified in this study was derived using the Youden index and evaluated in the same cohort. It should therefore be regarded as an exploratory, cohort-derived threshold rather than a universally applicable clinical cutoff. Differences in assay methodology, sample preparation, and patient populations may result in different optimal thresholds in other settings.

An important observation in our study was the discordance between serum CP and the established serum biomarkers. In 8 patients with PJI, serum CP was above the cohort-derived threshold while both CRP and IL-6 remained below their respective thresholds. This finding raises the possibility that CP may provide inflammatory information that is not completely captured by CRP or IL-6. However, the number of such cases was small, and these findings should therefore be considered hypothesis-generating rather than evidence of clinically meaningful incremental diagnostic value.

Conversely, in 10 patients with PJI, serum CP remained below the threshold despite elevated CRP and IL-6. This finding further emphasizes that CP does not identify all PJI cases detected by established biomarkers and supports its consideration as a potential adjunct rather than a replacement for CRP or IL-6.

The present study was not designed to establish a specific clinical scenario in which CP is preferable to CRP or IL-6, and no formal incremental diagnostic value analysis was performed. Accordingly, our findings are insufficient to recommend serum CP for treatment decisions or surgical planning on its own. Prospective studies incorporating formal measures of incremental diagnostic value will be necessary to determine whether CP adds clinically relevant information to existing PJI diagnostic algorithms.

In our cohort, serum CP levels did not differ significantly between patients with THA and TKA. Similar diagnostic performance was also observed in the separate THA and TKA analyses. These findings suggest that serum CP may have broadly comparable diagnostic characteristics in hip and knee arthroplasty patients. However, these subgroup analyses were exploratory, and the study was not specifically designed or powered to establish equivalence between the 2 arthroplasty types.

In addition to evaluating the diagnostic performance of serum CP, this study explored the potential influence of demographic factors, including age, gender, and type of joint (THA vs TKA), on serum CP levels. Our analyses showed no significant differences in median serum CP levels between men and women or between patients with hip vs knee arthroplasties, suggesting that these factors do not substantially affect CP measurement.

Age was associated with PJI prevalence, as patients with PJI were on average older than those without infection. However, age did not correlate significantly with serum CP levels (rho = 0.075, *P* = .134), and subgroup analyses comparing patients aged ≥70 years vs <70 years showed no statistically significant difference in CP values. These findings suggest that serum CP concentrations were not substantially influenced by age within our cohort. Nevertheless, larger studies with more balanced age distributions are required to confirm these findings.

Both serum CRP and IL-6 yielded higher sensitivity and specificity than serum CP in this study. While both CRP and IL-6 are established biomarkers, serum CP is a relatively new marker for diagnosing PJI.

The lower diagnostic performance of CP compared with CRP and IL-6 may partly reflect differences in assay methodology. In the present study, serum CP was measured using an immunoturbidimetric assay originally developed for fecal CP and adapted for serum samples by 1:10 dilution. The assay has not been formally validated by the manufacturer for serum. Differences in analytical platforms, sample preparation, and assay characteristics may therefore influence absolute CP concentrations and diagnostic thresholds.

Interestingly, the sensitivity and specificity observed for CRP and IL-6 in our cohort were also lower than those reported in previous meta-analyses [24], [25], [26]. However, this does not imply that serum CP would necessarily perform better under more controlled conditions. Differences between studies may reflect variations in patient populations, diagnostic definitions, assay methods, and disease spectrum. Therefore, the diagnostic performance of serum CP should be evaluated independently in prospective and externally validated cohorts rather than inferred from comparisons with published performance estimates of other biomarkers.

While serum CP levels might be influenced by several limiting factors in this study, one key advantage of serum CP is its apparent resilience to interference from noninflammatory conditions [27], [28], especially in the early postoperative period, where CRP is known to be elevated independently from possible PJI [29]. However, the present study did not specifically evaluate patients according to the interval from arthroplasty or systematically investigate early postoperative PJI. Therefore, a potential advantage of CP in this setting remains speculative and requires prospective evaluation.

Several limitations of this study should be considered. Partial verification bias cannot be completely excluded because the reference standard was not applied uniformly across the entire cohort. Synovial aspiration was not routinely performed in patients with a low preoperative probability of PJI, while intraoperative tissue sampling was only available in patients undergoing revision surgery. Nevertheless, all patients classified as having PJI underwent microbiological assessment, and sensitivity analyses restricted to patients with microbiological or intraoperative sampling yielded broadly comparable diagnostic performance estimates. These findings suggest that the primary results were relatively robust to differences in the diagnostic pathway. However, occult low-grade infections in patients who did not undergo aspiration or revision surgery cannot be completely excluded.

The interval between the index arthroplasty and diagnostic evaluation was not systematically recorded. Consequently, patients could not be stratified according to early postoperative vs late presentation, and the potential influence of time since arthroplasty on the diagnostic performance of serum CP, CRP, and IL-6 could not be assessed. This is particularly relevant because inflammatory markers may be affected by the normal postoperative inflammatory response. Although serum CP may theoretically be less susceptible to such non-specific postoperative inflammation than CRP [27], [28], [29], this potential advantage could not be evaluated in the present cohort and therefore remains speculative. Prospective studies with predefined postoperative intervals are required to address this issue.

The analytical methodology used for serum CP measurement also warrants consideration. The fCAL turbo assay was originally developed for fecal CP and was adapted for serum analysis using a 1:10 dilution. Because this application has not been formally validated by the manufacturer for serum samples, its analytical performance may differ from that of assays specifically developed and validated for serum CP. Differences in sample handling, dilution procedures, analytical platforms, and assay characteristics may therefore limit inter-institutional comparability of absolute serum CP concentrations and cut-off values. Accordingly, the cutoff identified in the present study should be regarded as assay- and laboratory-specific.

Finally, the cut-off values for serum CP, CRP, and IL-6 were derived using the Youden index and evaluated within the same study cohort. The resulting thresholds should therefore be considered exploratory and require validation in independent, preferably prospective cohorts before clinical implementation.

## Conclusions

Serum CP demonstrated moderate diagnostic performance for PJI, with lower overall diagnostic discrimination than CRP and IL-6. Although CP may provide complementary information in selected patients, particularly in cases with discordant conventional serum biomarkers, its incremental diagnostic value was not established in this study. Prospective studies with independent validation are required to determine whether serum CP provides clinically relevant additional information within established PJI diagnostic algorithms.

## Declaration of generative AI and AI-assisted technologies in the writing process

During the preparation of this work, the author(s) used perplexity AI and Chat-GPT-5 (OpenAI) in order to check grammar, spelling and to receive suggestions for wording and translation. After using this tool/service, the author(s) reviewed and edited the content as needed and take(s) full responsibility for the content of the published article.

## Funding

The open access publication costs are covered by the Elsevier-DEAL agreement with the University of Münster.

## Conflicts of interest

C. Theil receives honoraria for speakers’ bureau activities or paid presentations from Implantcast GmbH (Buxtehude, Germany) and research support as a Principal Investigator from Stiftung Endoprothetik (Hamburg, Germany); J. Schwarze receives other financial or material support and travel expenses from Implantcast GmbH; all other authors declare no potential conflicts of interest.

For full disclosure statements refer to https://doi.org/10.1016/j.artd.2026.102194.

## CRediT authorship contribution statement

**Morlin Hannah Schelling:** Writing – original draft, Visualization, Methodology, Investigation, Formal analysis, Data curation, Conceptualization. **Jan Schwarze:** Writing – review & editing, Conceptualization. **Georg Gosheger:** Writing – review & editing, Conceptualization. **Kristian Nikolaus Schneider:** Writing – review & editing, Conceptualization. **Sebastian Bockholt:** Writing – review & editing, Conceptualization. **Christoph Theil:** Writing – review & editing, Methodology, Investigation, Formal analysis, Data curation, Conceptualization.

## Appendix A Supplementary Data

Conflict of Interest Statement for Bockholt

Conflict of Interest Statement for Gosheger

Conflict of Interest Statement for Schelling

Conflict of Interest Statement for Schneider

Conflict of Interest Statement for Schwarze

Conflict of Interest Statement for Theil
