# Supplementary material for: Serum Calprotectin Demonstrates Moderate Diagnostic Performance for Detecting Periprosthetic Joint Infection in Hip and Knee Arthroplasty
Source: Arthroplast Today. 2026 Nov 3;42:102194. doi: 10.1016/j.artd.2026.102194 (PMC13756301; doi:10.1016/j.artd.2026.102194)
Supplement: Supplementary file 2 [file mmc2.pdf]

INDIVIDUAL CONFLICT OF INTEREST STATEMENT

American Association of Hip and Knee Surgeons

(Adopted from the American Academy of Orthopaedic Surgeons disclosure statement)

The following form **must be filled out completely and submitted by each author (example, 6 authors, 6 forms). All items require a response. If there is no relevant disclosure for a given item, enter "*None.*"**

Manuscript Title: Moderate sensitivity of serum calprotectin in the diagnosis periprosthetic joint infection

---

1. Royalties from a company or supplier (The following conflicts were disclosed)
None.

2. Speakers bureau/paid presentations for a company or supplier (The following conflicts were disclosed)
None.

3A. Paid employee for a company or supplier (The following conflicts were disclosed)
None.

3B. Paid consultant for a company or supplier (The following conflicts were disclosed)
None.

3C. Unpaid consultants for a company or supplier (The following conflicts were disclosed)
None.

4. Stock or stock options in a company or supplier (The following conflicts were disclosed)
None.

5. Research support from a company or supplier as a Principal Investigator (The following conflicts were disclosed)
None.

6. Other financial or material support from a company or supplier (The following conflicts were disclosed)
None.

7. Royalties, financial or material support from publishers (The following conflicts were disclosed)
None.

8. Medical/Orthopaedic publications editorial/governing board (The following conflicts were disclosed)
None.

9. Board member/committee appointments for a society (The following conflicts were disclosed)
None.

Each author must sign AND print or type his/her name, date and submit a separate form

In addition, one BLINDED Conflict of Interest form (no author names used) should be submitted per manuscript with all author disclosures.

GOSHEGER, GEORG | [signature] | 02/17/26

Author Name (Print or Type) | Author Signature | Date
