# Supplementary material for: Serum Calprotectin Demonstrates Moderate Diagnostic Performance for Detecting Periprosthetic Joint Infection in Hip and Knee Arthroplasty
Source: Arthroplast Today. 2026 Nov 3;42:102194. doi: 10.1016/j.artd.2026.102194 (PMC13756301; doi:10.1016/j.artd.2026.102194)
Supplement: Supplementary file 5 [file mmc5.pdf]

INDIVIDUAL CONFLICT OF INTEREST STATEMENT

American Association of Hip and Knee Surgeons

(Adopted from the American Academy of Orthopaedic Surgeons disclosure statement)

The following form **must be filled out completely and submitted by each author (example, 6 authors, 6 forms). All items require a response. If there is no relevant disclosure for a given item, enter "*None*."**

Manuscript Title: **Moderate sensitivity of serum calprotectin in the diagnosis periprosthetic joint infection**

---

1. Royalties from a company or supplier (The following conflicts were disclosed)
   None.

2. Speakers bureau/paid presentations for a company or supplier (The following conflicts were disclosed)
   None.

3A. Paid employee for a company or supplier (The following conflicts were disclosed)
   None.

3B. Paid consultant for a company or supplier (The following conflicts were disclosed)
   None.

3C. Unpaid consultants for a company or supplier (The following conflicts were disclosed)
   None.

4. Stock or stock options in a company or supplier (The following conflicts were disclosed)
   None.

5. Research support from a company or supplier as a Principal Investigator (The following conflicts were disclosed)
   None.

6. Other financial or material support from a company or supplier (The following conflicts were disclosed)
   Travel expenses from implantcast GmbH.

7. Royalties, financial or material support from publishers (The following conflicts were disclosed)
   None.

8. Medical/Orthopaedic publications editorial/governing board (The following conflicts were disclosed)
   None.

9. Board member/committee appointments for a society (The following conflicts were disclosed)
   None.

Each author must sign AND print or type his/her name, date and submit a separate form

In addition, one BLINDED Conflict of Interest form (no author names used) should be submitted per manuscript with all author disclosures.

Jan Schwarze	[signature]	18.02.2026
Author Name (Print or Type)	Author Signature	Date
